# Microbial Growth and Carbon Use Efficiency in the Rhizosphere and Root-Free Soil

**DOI:** 10.1371/journal.pone.0093282

**Published:** 2014-04-10

**Authors:** Evgenia Blagodatskaya, Sergey Blagodatsky, Traute-Heidi Anderson, Yakov Kuzyakov

**Affiliations:** 1 Soil Science of Temperate Ecosystems, Büsgen-Institute, University of Göttingen, Göttingen, Germany; 2 Institute of Physicochemical and Biological Problems in Soil Science, Russian Academy of Sciences, Pushchino, Russia; 3 Agricultural Soil Science, Büsgen-Institute, University of Göttingen, Göttingen, Germany; 4 Institute for Plant Production and Agroecology in the Tropics and Subtropics, University of Hohenheim, Stuttgart, Germany; 5 Thünen-Institute of Climate-Smart Agriculture (vTI), Braunschweig, Germany; University of Massachusetts, United States of America

## Abstract

Plant-microbial interactions alter C and N balance in the rhizosphere and affect the microbial carbon use efficiency (CUE)–the fundamental characteristic of microbial metabolism. Estimation of CUE in microbial hotspots with high dynamics of activity and changes of microbial physiological state from dormancy to activity is a challenge in soil microbiology. We analyzed respiratory activity, microbial DNA content and CUE by manipulation the C and nutrients availability in the soil under *Beta vulgaris*. All measurements were done in root-free and rhizosphere soil under steady-state conditions and during microbial growth induced by addition of glucose. Microorganisms in the rhizosphere and root-free soil differed in their CUE dynamics due to varying time delays between respiration burst and DNA increase. Constant CUE in an exponentially-growing microbial community in rhizosphere demonstrated the balanced growth. In contrast, the CUE in the root-free soil increased more than three times at the end of exponential growth and was 1.5 times higher than in the rhizosphere. Plants alter the dynamics of microbial CUE by balancing the catabolic and anabolic processes, which were decoupled in the root-free soil. The effects of N and C availability on CUE in rhizosphere and root-free soil are discussed.

## Introduction

Analysis of microbial carbon use efficiency (CUE) and microbial turnover rates are critical for accounting of C balance in soil with the goal of correct estimation of C sequestration potential as well as for modelling the turnover of soil C and CO_2_ fluxes [1]–[3]. The efficiency of microbial growth on a carbonaceous substrate coming with plant residues is positively related to formation rates of soil organic carbon [4]. A magnitude and dynamics of CUE is a function of numerous physical, chemical and ecological factors, e.g. soil quality [5], microbial community composition [6], [7], substrate and nutrient availability [3], [8], etc. At that the factor specific mechanisms, which control the CUE, remain uncertain [9]. This calls for the case studies under control conditions, so that the number of influencing factors can be reduced. So, preferential objects for CUE studies are the soils similar in physico-chemical characteristics but contrasting in substrate availability: e.g. rhizosphere and root-free soil. Higher microbial abundance and diversity and faster microbial growth occur in the rhizosphere soil as compared to root-free soil [10], [11] due to the high availability of C exuded by roots [1], [12]. Contrary to this, permanent limitation by available substrates in root-free soil leads to the selection of microorganisms with slower growth rates and more efficient metabolism [13]. So, rhizosphere and root-free soil can serve as good model for an *in situ* comparison of microbial physiology and CUE in microhabitats with contrasting resource levels.

CUE has become a very popular but ambiguous term in soil science. It is often used with a broad meaning, combining the efficiency of growth and the efficiency of maintenance of soil microorganisms [3]. Here, we introduce basic terms and approaches applicable either for distinct growth or for sustaining microbial biomass.

### CUE Estimation for Growing Microbial Biomass

During microbial growth, CUE is equivalent to the microbial yield coefficient (*Y*, g C_mic_ g^−1^ C_s_), i.e. biomass-C increment per amount of substrate-C used (Eq. 1, [14]):
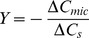
(1)where Δ*C_mic_* is the increase in microbial biomass-C caused by the consumption of substrate-C Δ*C_s_*. So, for **estimation of CUE for growing microbial biomass,** we used the microbial yield coefficient (Y). In spite of wide variability of the experimental Y estimations in the range of 0.1 to 0.8 [6], [15], [16] and a maximal theoretical value of 0.62 for glucose [17], the fixed value of Y = 0.45 is often assumed in soil studies and models [1], [18]. Considering very high variation (about 8 times) such a rough overall assumption of the average of 0.45 applied for different soils can distort the estimations and predictions of C stocks and fluxes [5], [18].

### CUE Estimation under Steady-state Conditions

In the absence of microbial growth, the estimation of Y (Eq. 1) is not applicable. However, even without distinct exponential growth, the substrate can be used both for maintenance and for the very slow, “cryptic” growth [19], so that microbial biomass does not decrease in time. Under such steady-state conditions, the estimation of the efficiency of microbial metabolism by specific respiration (CO_2_ produced per time and microbial biomass unit) can be used as a physiological characteristic.

The dormancy or maintenance state of microbial community reveals itself as a low respiration-to-biomass ratio which has been suggested as a physiological index of soil microbial communities [20]. The maintenance requirements are higher for microorganisms adapted to permanent input of available substrates than for microbial communities from nutrient-limited microhabitats [21]. The similar relationship is valid for growth expenses: the amount of respired CO_2_ during growth is larger for microbial communities with a higher growth rate and comparatively less efficient metabolism [22]. So, we hypothesised that both in the presence and absence of an available substrate, microbial communities in rhizosphere soil will have higher specific respiration rates than those in root-free soil.

### CUE Estimation during Shift from Dormancy to Active Stage

It is important to consider the CUE not only as a growth parameter (Y) and as a dormancy characteristic (maintenance coefficient), but also as the amount of CO_2_ produced per biomass unit in the course of the famine-to-feast transition. How such a transition alters CUE dynamics under changing environmental conditions, i.e. from substrate-limited to substrate-rich microhabitats, remains unclear. In contrast to steady-state or growth conditions where CUE remains constant, the experimental estimation of CUE during the famine-to-feast microbial transition remains a challenge for environmental microbiology. This is because the application of standard methods (fumigation-extraction or substrate-induced respiration) is restricted for biomass assessment in growing microbial communities.

A strong positive correlation between DNA and microbial C in soil [5], [23]–[25] led us use the DNA content as a proxy of microbial biomass. The increase in microbial DNA content corresponds to the respiratory response during exponential microbial growth after substrate addition [24], [26]. Therefore, we used the CO_2_/DNA ratio for comparison of the CUE by transition from dormant to active stage for microbial communities with contrasting growth strategies. Experimentally, the growth strategies can be evaluated by the maximal specific growth rate under unlimited conditions that is greater for *r*- than for *K*-strategists [27], [28]. So, we used two parameters of microbial metabolism: microbial maximal specific growth rates and CUE, to evaluate the relative abundance of slow- or fast-growing microorganisms in rhizosphere and root-free soil.

### Nitrogen Effect on CUE

The efficiency of microbial metabolism depends strongly on nitrogen (N) availability [29]. Lower respiration due to higher efficiency of microbial C reutilisation has been observed in the absence of N limitation as compared to N-limited conditions [30]. Nitrogen addition reduces cumulative microbial respiration in soil amended with glucose [31] and plant litter [32] and increased the growth yield efficiency [18]. While the CUE decline under N limitation is commonly expected [3], it is unknown whether N availability affects equally microbial respiration and growth rates in microhabitats with contrast substrate availability, e.g. in root-free and rhizosphere soil [33]. Therefore, we compared the specific respiration and microbial growth kinetics in the root-free and in rhizosphere soil with different N fertilization rates. We expected to find more distinct effect of N availability in the rhizosphere where microbial activity and abundance are higher and N limitation may be more important as compared to root-free soil. We hypothesized that the increase of N availability improves CUE and decreases specific respiration, especially in the rhizosphere.

We analyzed the ratio between respiration and microbial DNA content 1) under steady state conditions (in unamended soil), 2) during microbial growth in soil amended with glucose, and 3) during transition from steady state conditions to growth. In addition, effect of N availability on microbial growth rate and CUE was determined. Three complementary indices were applied as indicators of the efficiency of microbial metabolism in the rhizosphere and in root-free soil: 1) the CO_2_ */DNA* ratio further referred to as ‘specific respiration rate’, 2) the ΔCO_2_/ΔDNA ratio for growing biomass, and 3) CUE during microbial growth on glucose.

## Materials and Methods

### Soil Sampling

Soil samples were taken from the field experimental station at the Institute of Agroecology (FAL, Braunschweig, Germany). No specific permission was required as one of the co-authors (THA) had been working in the Institute of Agroecology, and soil was regularly sampled in the course of long-term field trial described elsewhere [34]. The soil is a loamy sand Haplic Cambisol (C_org_ 1.1%; N_tot_ 0.087%; pH_CaCl2_ 6.7). The plots under sugar beet (*Beta vulgaris* subsp. *rapacea* (KOCH-DÖLL, cv. Wiebke) with full and half the recommended rate of mineral N fertiliser (126 and 63 kg N ha^−1^ year^−1^, respectively) were chosen for analysis of the N effects on microbial communities of root-free and rhizosphere soil. Soil was sampled during harvesting the sugar beet at a mature stage (age 4.5-month). Soil samples were taken from the 0–10 cm layer from five randomly chosen replicate microsites and then mixed. Rhizosphere soil was sampled at a distance 1–5 mm adjacent to the roots (i.e. collecting the soil aggregates falling off when shaking the root system), whereas root-free soil was taken between rows of sugar beets. Fine roots and other plant debris were carefully removed during sampling. No significant differences were detected in pH, C_t_ or N_t_ content of the rhizosphere and root-free soil. The soil was stored field-fresh in aerated polyethylene bags at 4°C for 1–2 weeks. Prior to analysis the soil was sieved (<2 mm), moistured to 60% of WHC, and preincubated at 22°C for 24 h.

### Soil Respiration and Chemical Analysis

Microbial biomass (C_mic_) was determined by the initial rate of substrate-induced respiration after soil amendment with glucose and according to the equation of Anderson & Domsch [35]:
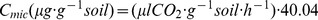
(2)


Rate of basal respiration (V_basal_) was estimated for soil without glucose as the hourly mean of 10 h of CO_2_ evolution at 22°C, after 2–3 hours diminishing of the initial CO_2_ flush caused by soil disturbance during sample preparation [36]. The CO_2_ emission rate (V_CO2_) was measured hourly at 22°C using an automated infrared-gas analyser system [37].

Soil organic C and total N were analysed by dry combustion (C-IR 12, Leco, and Macro-N, Hereaus, respectively). Soil pH was measured in 0.01 M CaCl_2_ with a soil-to-solution ratio of 1∶2.

### Total DNA

Quantity of double-stranded DNA was determined by direct DNA isolation from the soil with mechanic and enzymatic disruption of microbial cell walls and subsequent spectrofluorimetric detection with PicoGreen [23], [24]. For rhizosphere and root-free soil from plot fertilized with 126 kg N ha^−1^ year^−1^ the dsDNA determination was done at 0, 12, 15, 20, 25 and 36 hours after addition of glucose and nutrients (as described below for respiration kinetics).

The procedure of DNA isolation involved sonication of the soil suspension in Tris-EDTA buffer (TE) at pH 8, addition of aurintricarboxilic acid (a nuclease inhibitor) and sodium dodecyl sulphate. Then two cycles of quick freeze at −80°C in Deep Freezer (ProfiMaster EPF3080/N, National Lab GmbH, Mölln, Germany) for 1 h and subsequent thaw at +65°C in water bath with thermostat (Model 1002, GFL Gesellschaft für Labortechnik mbH, Burgwedel, Germany) were performed to destroy microbial cells. Enzymatic digestion was accomplished with lysozyme and Proteinase K for 1 h at 37°C. Mechanical destruction of microbial cells was implemented by shaking with sterile acid-washed glass-beads (Sigma-Aldrich, Inc.) of three sizes (710–1180, 212–300, and <106 μm) on a Vortex homogeniser at 2000 rpm. The samples were diluted with an equal volume of TE-buffer and centrifuged for 10 min at 5500 g. Half a millilitre of the diluted supernatant (1∶100) was mixed with 0.5 ml of a 1∶200 dilution of PicoGreen™ (Molecular Probes). After 4 min incubation, the fluorescence was measured on an SFM-25 spectrofluorimeter (Kontron, Germany) at an excitation wavelength of 480 nm and an emission wavelength of 523 nm. The dsDNA of bacteriophage lambda was used as a standard; samples for the standard curve were prepared in TE-buffer in the same way as the experimental samples.

### Kinetic Parameters of Microbial Growth

Kinetics of microbial growth was determined indirectly by the rate of CO_2_ emission from soil amended with glucose and mineral nutrients [38]. It has to be noted that despite substrate addition is required for the estimation of kinetic parameters (specific growth rate, active and total microbial biomass, see below), the results obtained by this approach (substrate induced growth response – SIGR) are the characteristics of the soil microbial community at the sampling instant, i.e. before substrate addition. Samples of 10 g (dry weight) soil were amended with a powder-mixture containing glucose (10 mg g^−1^), talcum (20 mg g^−1^) and mineral salts: (NH_4_)_2_SO_4_−1.9 mg g^−1^, K_2_HPO_4_−2.25 mg g^−1^ and MgSO_4_·7H_2_O−3.8 mg g^−1^ [39]. These optimal concentrations of the substrates were selected in preliminary experiments and are sufficient for unlimited exponential growth of soil microorganisms at least during several hours needed for recording of respiration kinetics. Mineral salts were chosen considering the pH value and buffer capacity of the soil so that the pH was not changed more than 0.1 pH units. Soil samples were placed (in triplicate) in an ADC2250 24-channel Soil Respiration System (ADC Bioscientific, Herts, UK) at 22°C. Each sample was continuously aerated (300 mL min^−^1), and the rate of CO_2_ production from each sample was measured every hour using an infrared detector and mass-flow meter [37].

*Maximal specific microbial growth rate* (μ_m_) was determined by fitting the model parameters to the measured data on CO_2_ production:
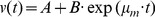
(3)where ***ν***(***t***) - CO_2_ evolution rate at time (t), ***A*** - initial rate of uncoupled (non-growth) respiration, ***B*** - initial rate of coupled (growth) respiration [19], [40]. Fitting was restricted to the initial phase of the curve, which corresponded to unlimited exponential growth [41]. Maximum values of statistic criteria: r^2^, the fraction of total variation explained by the model were used for fitting optimisation. Further goodness of fit estimations were made and based on the Q value derived from *χ* ^2^ [42].

Activity status of the microbial biomass r_0_ was calculated from the ratio of A:B [19]:
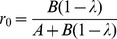
(4)where λ may be accepted as a basic stoichiometric constant = 0.9 [19]. The total glucose-metabolizing microbial biomass (sustaining + growing; x_0_) was calculated as following:
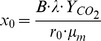
(5)where Y_CO2_ is yield of biomass C per unit of respired C-CO2.

The *growing microbial biomass* (x_0_′) was calculated using the equation:
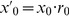
(6)


More complete theoretical background and details on equations derivation were described elsewhere [28], [38], [40].

The duration of lag-period (t_lag_) – a period characterised by stable respiration preceding microbial growth – was defined as the time from glucose addition to the time when the increasing rate of growth-associated respiration (B* exp(μ_m_ *t)) equalled the rate of non-growth respiration (***A***) [43]. The lag-period was calculated using parameters of Eq. 3:
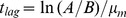
(7)


The ratio of *CO_2_ increment-to-DNA increment* (ΔCO_2_/ΔDNA) was calculated as the amount of CO_2_ in μg C evolved per μg of DNA increment during the same period. The amount of respired CO_2_ in soil amended with glucose was corrected for basal respiration, i.e. the corresponding amount of CO_2_ respired from the unamended soil during the same period was subtracted from the CO_2_ increment for glucose-amended soil.

The carbon use efficiency or CUE (in the growth phase, this is equivalent to the growth yield quotient, Y, Eq.1) was calculated as biomass C increment per amount of consumed C-substrate, which is in turn equal to biomass C increment plus CO_2_ evolved:
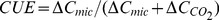
(8)where ΔC_mic_ is the net increase in microbial biomass C (μg C g^−1^) and ΔC_CO2_ is the net increase in cumulative respiration (μg C g^−1^) corrected for basal respiration. Microbial C content was calculated from mean measured DNA content found in our study (11% of dry biomass), assuming that the C content in microbial biomass is 45% [5], [44].

### Statistical Analyses

The means of three replicates with standard errors are presented in tables and figures. Two-way ANOVA was applied to characterise the effects of C and N availability: 1) C availability: rhizosphere versus root-free soil, and 2) N availability: half versus full N fertilisation. When significant effects were found, a multiple comparison using the Student-Newman-Keuls test (P<0.05) was performed. All variables passed normality and equal variance tests.

## Results

### Basal Respiration Rate, DNA Content and Microbial Biomass

**Figure 1:**
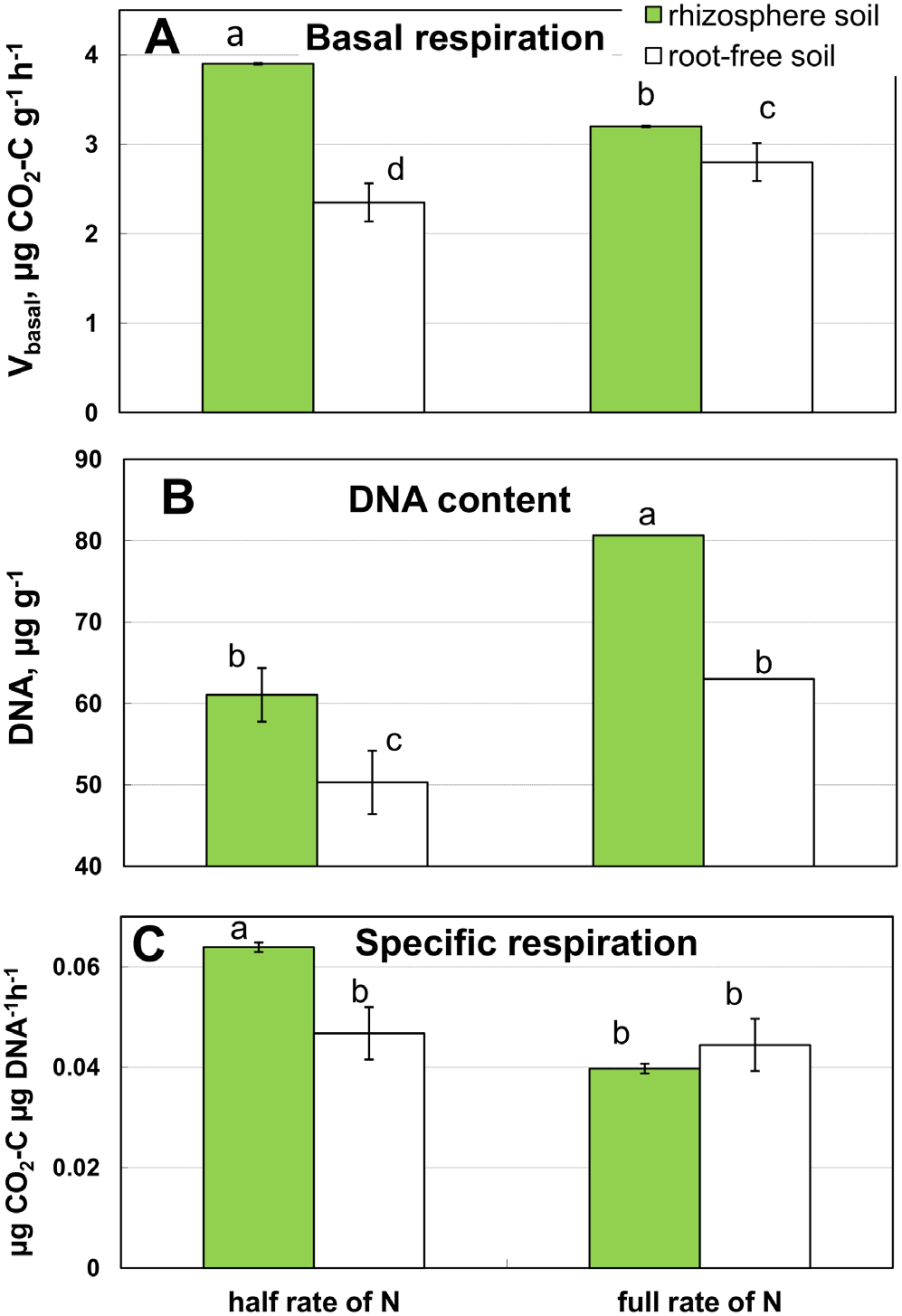
Respiration rate and microbial DNA in soil and rhizosphere. Basal respiration rate (a), microbial DNA content (b), and ratio of basal respiration rate (V_basal_) to DNA content (c) of rhizosphere and root-free soil under *Beta vulgaris* at half (63 kg N ha^−1^) and full (126 kg N ha^−1^) rates of nitrogen fertilisation.

The basal respiration rate (V_basal_) was significantly higher in the rhizosphere as compared to root-free soil (Fig. 1a). This rhizosphere effect amounted to 66% at the half N rate while it was only 14% at the full rate of N application. The V_basal_ in root-free soil was significantly higher at the full versus half rate of N-fertilisation (Fig. 1a). In rhizosphere soil, however, N fertilisation significantly decreased basal respiration.

Microbial DNA content was higher at the full N rate than in the corresponding treatments with the half N (Fig. 1b). Higher DNA content in rhizosphere versus root-free soil (28% at the full and 21% at the half N rate) reflects a pronounced rhizosphere effect.

**Figure 2:**
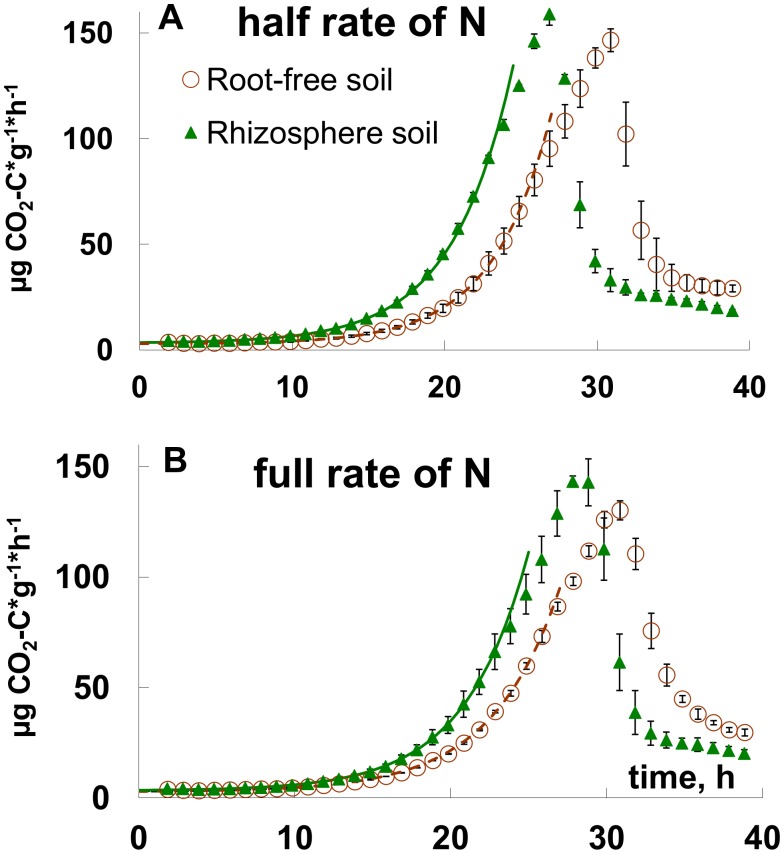
Dynamics of microbial respiration after glucose addition to root-free and rhizosphere soil. Glucose and nutrients induced respiration rate in root-free and rhizosphere soil under *Beta vulgaris* at half (a) and full (b) rates of N fertiliser. Experimental points and curves fitted by Eq. 3 for unlimited growth period are presented.

Microbial respiration curves during growth on glucose were clearly different between the rhizosphere and root-free soil (Fig. 2). These differences were more pronounced under N limitation (Fig. 2). Maximal specific growth rates (**μ_m_**) were significantly higher, while the duration of the lag-period was 1.7–1.9 h shorter in the rhizosphere than in root-free soil (Table 1).

**Table 1:** Biomass and kinetic parameters of the respiratory response of microorganisms growing on glucose.

		Microbial biomass C			Total cell mass	Maximal growth rate (μ_m_)	Lag-period (t_lag_)
		Total	Growing	Growing			
Soil	N rate	μg C g^−1^	μg C g^−1^	% of total	μg g^−1^	h^−1^	h
Root-free	50%	221^c^±1	0.486^c^±0.04	0.22	491±2	0.250^b^±0.003	12.2^a^±0.3
Rhizosphere	50%	290^a^±20	0.888^a^±0.02	0.31	644±44	0.260^a^±0.001	10.3^b^±0.2
Root-free	100%	245^b^±14	0.637^b^±0.03	0.26	544±31	0.238^c^±0.002	12.4^a^±0.2
Rhizosphere	100%	280^a^±1	0.922^a^±0.05	0.33	622±2	0.246^b^±0.002	10.7^b^±0.5

Total cell mass was calculated assuming a C content of the microbial biomass of 45% of dry weight (Christensen et al., 1993). Small letters show significant differences within the same column (p<0.05).

Both the total microbial biomass C and its growing fraction were always higher in the rhizosphere as compared to root-free soil (Table 1). This rhizosphere effect was most pronounced at half versus the full N rate (Table 1) and amounted to 31% and 14% of the total microbial biomass, respectively. Actively growing microbial biomass did not exceed 0.34% of total microbial C and was much more sensitive to the presence of roots as compared to total microbial biomass. So, the rhizosphere effect for growing microbial biomass was much greater than for the total microbial biomass and amounted to 45% at full N and to 83% at the half N rate (Table 1). The direct effect of N on total microbial biomass was insignificant in rhizosphere soil, while in root-free soil significantly higher microbial biomass C was observed at the full N rate.

Two-way ANOVA confirmed the strong effects of roots of *Beta vulgaris* on all microbial parameters tested (Table 2). The portion of active microbial biomass and the lag-period were affected by roots at the largest extent: more than 90% of their variation was explained by the rhizosphere effect. The direct effect of N on the specific growth rate (**μ_m_**) and DNA was even stronger than the effect of roots (Table 2).

**Table 2:** Contribution of two factors: living roots (Roots) and N fertilisation rate (N) and their interactions (Roots x N) to the variance of microbial parameters.

Factor	Basal	Microbial biomass		dsDNA	Maximal	Lag-period
	respiration	total	active	content	growth rate, μ_m_	
Roots	67.2***	86.7***	89.8***	40.7***	30.6**	95.1***
N	0.6^ns^	1.5^ns^	6.7**	48.1***	63.8**	1.7^ns^
Roots x N	28.6**	8.5*	2.5*	7.5***	0.4^ns^	0.3^ns^
Residual	3.9	3.3	1	3.7	5.2	2.9

two-way ANOVA, % of explained variance.

***, **, * - significant effects at P<0.001, <0.01 and <0.05, respectively.

ns – not significant.

We conclude that significantly higher basal respiration, DNA content and total and actively growing microbial biomass were observed in the rhizosphere versus root-free soil and this effect was more pronounced under low N fertilization.

### Respiratory Activity in Relation to DNA Content in Rhizosphere and Root-free Soil

The CO_2_/DNA ratio in the non-growing microbial community varied between 0.038 and 0.064 μg CO_2_-C μg^−1 ^DNA h^−1^ (Fig. 1c). The rhizosphere effect on the CO_2_/DNA ratio was significant only at the half N rate (Fig. 1c). A significant N effect was observed only in rhizosphere soil: the CO_2_/DNA ratio was 64% greater at the half versus the full N rate (Fig. 1c).

### Respiratory Response and Microbial DNA Dynamics during Glucose-induced Growth

**Figure 3:**
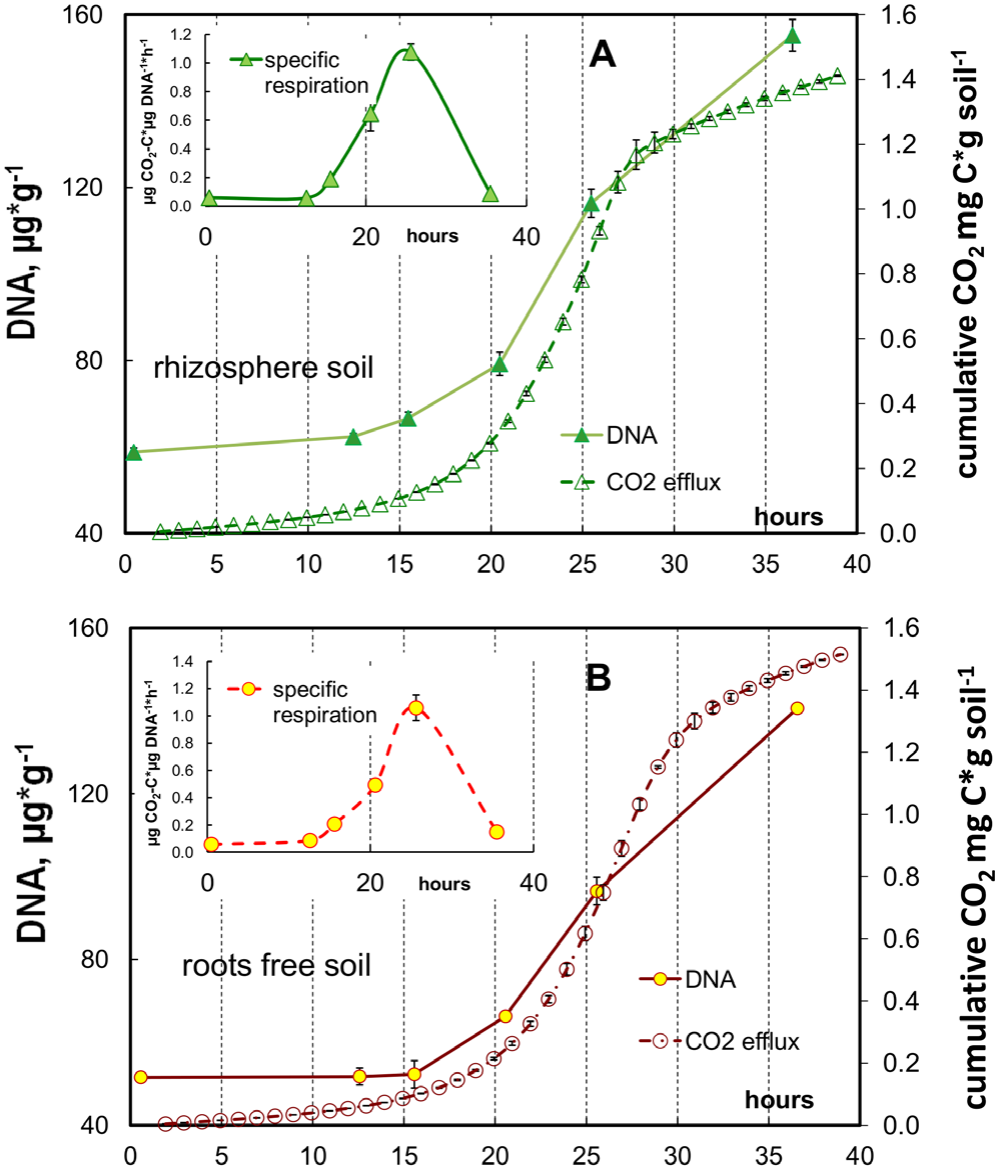
Microbial DNA dynamics and cumulative CO_2_ production in root-free and rhizosphere soil. Dynamics of microbial DNA content and CO_2_ accumulation after glucose addition in rhizosphere (a) and root-free (b) soil collected from the plot fertilized with 126 kg N ha^−1^ year^−1^. Dynamics of specific CO_2_ production (V_CO2_-to-DNA ratio) are shown in the inserted graphs for rhizosphere and root-free soil, correspondingly.

According to respiratory kinetics, we defined three phases of microbial growth on glucose (Fig. 2): an initial phase corresponding to the absence of microbial growth lasting in rhizosphere soil between 0 and −10.7 h (Table 1, see lag period); followed by the phase of exponential growth to 25.5 h; and by the phase of growth retardation thereafter. In root-free soil duration of corresponding microbial growth phases was for ca. 2 h (lag-phase) and even for 4 h longer than in the rhizosphere (Table 1, Fig. 2). The DNA content in the rhizosphere significantly increased within two hours after the end of the lag-period (t_lag_ 10.3 h, Tables 1, 3). Thus, the amount of DNA in the rhizosphere soil increased almost simultaneously with the respiration (Fig. 3a). In contrast, there were no changes in DNA content 15 hours after glucose application in root-free soil (Fig. 3b). So, contrary to the rhizosphere a time shift of at least three hours was observed between the increase of CO_2_ and of DNA.

**Table 3:** The amount of produced CO_2_, DNA increment and carbon use efficiency (CUE) at different phases of microbial growth after glucose addition.

Period after glucoseaddition, (h)	Location	Phase ofmicrobial growth	DNA increase during specified period	CO_2_ accumulated during specified period	CUE, calculated according Eq.8, see details in text
			μg g soil^−1^	μg C g soil^−1^	g C g C^−1^
0–12.5	Rhizosphere	lag-phase & initial growth	3.5±1.3	59^d^±3	0.41^a^±0.04
	Root-free soil	lag-phase	0.2±3.6	40^d^±2	0.39^a^±0.05
12.5–25.5	Rhizosphere	exponential growth	54.1±3.4	772^b^±22	0.23^b^±0.02
	Root-free soil	exponential growth	44.8±8.2	383^c^±39	0.35^a^±0.07
25.5–36.5	Rhizosphere	growth retardation growth	38.6±4.9	578^c^±26	0.22^b^±0.04
	Root-free soil	& growth retardation	43.5±8.5	877^b^±65	0.17^b^±0.06
0–36.5	Rhizosphere	all phases	96±3.8	1408^a^±1	0.23^b^±0.01
	Root-free soil	all phases	87.7±4.3	1300^a^±24	0.23^b^±0.02

Small letters show significant differences within the same column (p<0.05).

During the exponential growth, the specific rate of CO_2_ emission (V_CO2_ */DNA* ratio) steadily increased in both soils (Fig. 3 inserts). Despite the DNA content was significantly lower in root-free as compared to rhizosphere soil during the 35 h after glucose addition (Fig. 3), no significant differences (exception for one point at 20 h) between root-free and rhizosphere soil were found for the V_CO2_ */DNA* ratio, which peaked at 25 h after glucose addition and exceeded 1 μg C μg^−1 ^DNA h^−1^. After growth retardation, the V_CO2_ */DNA* ratios returned to the initial state and were close to 0.1 μg C μg^−1 ^DNA h^−1^ (Fig. 3 inserts).

**Figure 4:**
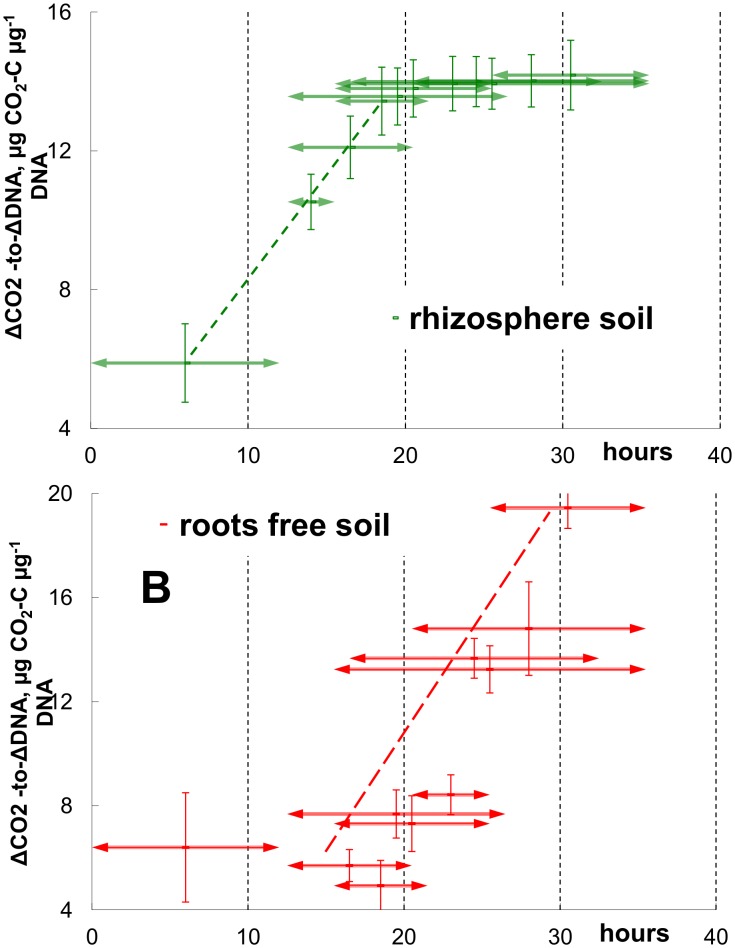
The ratio of CO_2_ increment-to-DNA increment in rhizosphere and root-free soil. Soil was collected from the plot fertilized with 126^−1^ year^−1^, (a) – rhizosphere soil, (b) – root-free soil. Horizontal arrows show the time period used for ΔCO_2_/ΔDNA ratio calculation. Vertical bars show standard deviations.

The quantity of CO_2_ evolved per unit of newly-formed DNA (ΔCO_2_/ΔDNA) from the rhizosphere soil continuously increased until the middle of the exponential growth, then stabilised until the end of incubation at 13.6±0.3 μg CO_2_-C μg^−1 ^DNA (Fig. 4a), indicating a proportional increase in CO_2_ and DNA content. In the root-free soil however, the ΔCO_2_/ΔDNA ratio was 1.5–2 times lower than in rhizosphere during exponential growth (until 20–23 h after glucose addition) and increased only after growth retardation (Fig. 4b). The microbial respiration rate decreased in the rhizosphere after 25 h, and in the root-free soil after 30 hours (Fig. 2), but the DNA content increased for at least 10 more hours in both soils (Fig. 3,). Twice as much CO_2_ was produced during exponential growth in rhizosphere versus root-free soil (Table 3), but only 8% more CO_2_ was evolved from rhizosphere as compared to root-free soil during the whole incubation (36 h after glucose addition). Thus, the more efficient growth in the exponential phase (according to the ΔCO_2_/ΔDNA ratio) was counterbalanced by a less efficient metabolism after substrate exhaustion in the root-free soil.

The CUE (Eq. 8) also indicated more efficient microbial metabolism in root-free versus rhizosphere soil during exponential growth (Table 3). At the early stage of glucose utilization and after growth retardation, however, the efficiency of microbial metabolism was lower in root-free than in rhizosphere soil. Remarkably, CUE estimated for the whole incubation period did not differ between both soils (Table 3).

## Discussion

### Microbial Biomass and DNA Content as a Basis for CUE Estimation

Assuming a C content of microbial biomass of 45% of dry weight [5], the total cell mass in soil without glucose varied from 491 to 644 μg g^−1^ soil (according to the SIR method, Eq. 2, Table 1). Therefore, the DNA content in microbial biomass amounted to 9.5–13% of dry weight which is in the upper range of the values reported for cultures extracted or isolated from soil bacteria, 5.2–13% [45] and is very close to the microbial DNA content *in situ* in soil (7–9%) when microbial biomass was assessed by a fumigation-extraction technique [26]. The comparison of several independent observations indicated that approximately 13% of the soil microbial biomass consisted of DNA [25]. However, the DNA content per biomass unit was not constant and decreased with increasing cell size from 13 to 5.2% [45] and was greater in non-growing than in growing bacterial cells. Therefore, the high DNA percentage in microbial biomass in our soil reflected the domination of small-sized cells in the non-growing microbial community.

### Respiration and DNA Content under Steady-state and Unlimited Growth Conditions

Our results (Fig. 3, insert) confirm the findings of Marstorp & Witter [26] for a sandy loam soil from central Sweden, where *CO_2_/DNA* ratios were lower than 0.1 μg CO_2_-C μg^−1 ^DNA h^−1^ for a non-growing microbial community. During exponential growth, however, we observed a quick increase in *CO_2_/DNA* ratios. The *CO_2_/DNA* ratio calculated according to Figure 1 in Marstorp & Witter [26] also increased during glucose-induced growth up to 0.5 μg CO_2_-C μg^−1 ^DNA h^−1^. The *CO_2_/DNA* ratio changed along with the physiological state of microorganisms and therefore, together with the metabolic quotient qCO_2_, can be used as a valuable ecophysiological indicator reflecting the activity status of microbial biomass in soil.

A constant DNA content during the lag-period has been observed for *in situ* soil conditions [26]. We noticed, however, that the increase in DNA content in root-free soil began several hours after the increase in respiration, reflecting a period necessary for the activation of microbial metabolism (CO_2_ increase) before the real growth (DNA increase) start. Such behaviour is common for *K-*strategists [46]. The delay between respiratory increase and DNA synthesis after the stimulation of microbial growth was much shorter in rhizosphere than in root-free soil, where no increase in DNA content was evident, even at the start of the exponential respiration increase. This was supported by the amount of active microbial biomass capable for immediate growth that was twice as large in rhizosphere as compared to root-free soil (Table 1).

We demonstrated two kinds of physiological responses to glucose addition in microbial communities in rhizosphere and root-free soil. The DNA synthesis after glucose addition was more closely coupled with CO_2_ production in rhizosphere soil as compared to root-free soil, where the dynamics of DNA synthesis and CO_2_ production were decoupled both immediately after glucose addition and after its exhaustion. Microorganisms in the root-free soil persisted in a dormant state and reacted to increased substrate availability with a distinct delay between respiration response and DNA synthesis. In the rhizosphere, where the fraction of active microorganisms capable for immediate growth was two-fold larger than in root-free soil, the microbial community responded to glucose earlier in terms of both respiration and DNA synthesis (Figs. 2, 3).

### Lag Period and Specific Growth Rates of Microorganisms in the Rhizosphere and Root-free Soil

The significantly greater μ_m_ values in rhizosphere as compared to root-free soil (Table 1) indicated a greater portion of fast growing microorganisms with r-strategy in the rhizosphere. Selective stimulation of some bacterial species in the rhizosphere (e. g. *Pseudomonas sp.*), [12], [47] with higher specific growth rates than most other soil bacteria [38] explains this phenomenon. The microbial community of the rhizosphere has a shorter lag-period and was ready for immediate growth on available substrate compared to the microbial community in root-free soil. According to Eq. 7, the duration of t_lag_ is dependent both on μ_m_ and on the fraction of actively growing microorganisms in the total microbial biomass. The negative correlation between lag-period and the amount of active biomass (r^2^ = −0.78, p<0.12) was stronger compared to correlation between t_lag_ and μ_m_ (r^2^ = −0.49, p<0.30). Thus, we conclude that the activity state of microbial biomass rather than such feature of the microorganisms as maximal specific growth rate (μ_m_) is responsible for the duration of t_lag_.

### Basal Respiration as a Response to N Limitation in Rhizosphere versus Root-free Soil

The inverse response of basal respiration rate to N fertilization level in the rhizosphere and root-free soil (Fig. 1a) reflected the different strategies of microbial growth in soil microhabitats. Microorganisms with r-strategy dominating in rhizosphere soil increased basal respiration under N limitation. This resulted in highest values of specific respiration (maintenance efficiency) and consequently in lowest CUE. Contrary to that, the K-strategists prevailing in root-free soil even decreased basal respiration in low N treatment, thus, maintaining CUE similar to that in high N plot under steady-state. There were no differences in fine root development between the plots with full and half rate of N at time of soil sampling [34]; therefore we do not attribute the observed differences in V_basal_ to the variation in C input from roots to the soil [48]. Double limitation by C and N in the root-free soil at the half N rate decreased both microbial DNA content and basal respiration compared to root-free soil at the full N rate. However, specific respiration (maintenance efficiency) did not differ significantly between half and full rate of N fertilization in root-free soil (Fig. 1c) demonstrating stronger competitive abilities of K-strategists under N limitation. Therefore, both the level of metabolic activity and CUE should be considered when the N effect on soil respiration is estimated.

### CUE in Rhizosphere and Root-free Soil: Dynamics and Proof of Estimates

Our study revealed the basic differences between microbial communities in rhizosphere and root-free soil in catabolic and anabolic processes traced by the dynamics of two fundamental microbial parameters: respiration activity (CO_2_) and cell proliferation (DNA), which were used for estimation of CUE. Lower CUE during exponential growth of the *r-*selected rhizosphere community (Table 3) was confirmed by the two-fold higher ΔCO_2_/ΔDNA ratios in rhizosphere versus root-free soil (Fig. 4, 15–20 hours). This agrees with the negative correlation between growth rate and yield [22], [49]. Contrary to *r-*strategists, the *K*-strategists relatively more abundant in root-free soil do not mineralise glucose immediately, but can partly store it as an intracellular reserve during lag-phase and use it later after substrate exhaustion [38], [50], [51], thus maintaining their respiratory activity longer. Remarkably, distinct differences in CUE between rhizosphere and root-free soil observed during exponential growth were completely smoothed for CUE estimated for the whole incubation period. Thus, the same energy input caused different patterns of catabolic and anabolic processes in r- and K-selected communities resulting in similar energy output per unit of newly formed DNA in rhizosphere and root-free soils. This demonstrates that the shift in balance between catabolic and anabolic processes can serve as a tool for microbial community to maintain CUE independently of changing environment.

The CUE estimated during the exponential growth was 22% and 35% for rhizosphere and root-free soil, respectively. This is close to the range of 20–30% found for a cultured population of indigenous soil bacteria in the growth phase [45] and it is in the range of 14–51% observed for 8 agricultural soils [5]. However, much higher CUE has been obtained by other methods for *in situ* microbial communities growing on ^14^C- or ^13^C-labeled glucose (50–61%, [30]; 69–78%, [18], see for review [3]).

We used the average DNA value of 11% of total microbial biomass that was determined in soil without glucose addition [5], [45]. Considering lower DNA content in growing cells versus the cells in stationary phase [45], and that the DNA content in fungal mycelium can be much lower than in bacterial cells [52], [53] the CUE of 38% and 51% can be obtained for rhizosphere and root-free soil, respectively (based on the lowest DNA content of 5.2% of cell mass for pure cultures [45]). These CUE exactly fits to the estimates for glucose use efficiency in N-amended and in N-limited soil (Y = 0.52 and 0.38, respectively) using a balance calculation [30]. The scatter of CUE values found in the literature can be explained by the variation in growth conditions of microorganisms affecting also the DNA content in microbial cells. More experimental studies on the variability of DNA content *in situ* are needed for narrowing CUE estimates in experiments similar to ours.

## Conclusions

The applied combination of approaches: analysis of the double-stranded DNA content in soil and of respiration kinetics allows quantitative distinguishing of microbial traits in the rhizosphere versus root free soil. Total microbial biomass in the rhizosphere was 14–31% higher than that in the root free soil, while the growing (active) part of microbial biomass was 45–83% higher. The higher microbial specific growth rate (μ_m_) and lower CUE indicated the greater contribution of *r-*strategists in rhizosphere as compared with root-free soil. We partly confirmed hypotheses posed in the introduction: microbial communities in rhizosphere soil have specific respiration rate higher than microorganisms in root-free soil. This holds true under N limiting conditions but no difference was observed for fully fertilized N plot. Lower content of available N decreased microbial DNA, but increased the μ_m_ values. The N limitation in the rhizosphere increased microbial respiration, presumably due to lower C use efficiency confirming domination of r-selected species in rhizosphere microbial community and supporting our second hypotheses.

The ΔCO_2_/ΔDNA ratio was stable in the growing microbial community in the rhizosphere while it increased consistently in root-free soil, revealing contrasting patterns of microbial metabolism in different microhabitats. The *K-*strategy typical for root-free soil manifested itself by decoupling of the respiration burst after glucose addition and DNA increase, more efficient growth (high CUE) and longer persistence of respiratory activity. The *r-*strategy (common for rhizosphere) was exhibited as a faster and simultaneous response on substrate addition, lower growth efficiency and a shorter period of high activity following by more abrupt respiration decrease after substrate exhaustion. The CUE during exponential growth was by the factor of 1.5 higher in root-free than in rhizosphere soil indicating the necessity to consider variable Y depending on substrate availability in soil microhabitats. Further studies are necessary for the determination of the range of differences in CUE in soil microhabitats, because microbial community composition depends on multiple factors such as host plant species, soil properties, plant development stage [10], [54] and these factors will affect also the microbial physiology in rhizosphere and root-free soils.
